# The impact of congenital heart disease on somatic growth: a monozygotic twin study

**DOI:** 10.1007/s00431-025-06635-2

**Published:** 2025-11-18

**Authors:** J. H. Lee, T. J. Yun, S. J. Kwon, Z. Troy, T. A. Marshall, H. G. Buck, P. J. Mulder, S. Daack-Hirsch

**Affiliations:** 1 Institute for Nursing and Interprofessional Research, Children’s Hospital Los Angeles, Los Angeles, CA USA; 2 College of Nursing, The University of Iowa, Iowa City, IA USA; 3 Division of Pediatric Cardiac Surgery, Asan Medical Center, University of Ulsan College of Medicine, Seoul, South Korea; 4 Congenital Heart Disease Center, Asan Medical Center, Seoul, Korea; 5 Preventive and Community Dentistry, The University of Iowa, Iowa City, IA USA

**Keywords:** Heart defects, Congenital, Twins, Failure to thrive, Malnutrition, Growth faltering

## Abstract

Children with congenital heart disease (CHD) often experience growth faltering. However, somatic growth is significantly influenced by genetic and environmental factors. Given the unique genetic and environmental similarities of monozygotic twins, this study aims to evaluate the relationship between CHD and growth faltering, while minimizing the influence of these factors. We retrospectively reviewed data from 41 pairs of twins, where one had CHD and the other did not. Anthropometric measures were collected from birth to 3 years of age. Growth differences within the CHD group were further analyzed based on disease characteristics, including cyanosis, pulmonary artery hypertension (PAH), heart failure (HF), and ventricular physiology. Mixed-effects regression models assessed longitudinal differences between twins, and multiple linear regression analyses identified predictors of anthropometric z-scores at 1 year. The CHD group showed a significant decline in all growth parameters from 1 year of age (*p* < .001), with no significant improvement during the observation period. Growth faltering was more common in the CHD group (46.3%) compared to the non-CHD group (7.5%). Within the CHD group, children with HF had significantly lower weight for age z-score and height for age z-score (*p* < .05). Being small for gestational age (SGA) independently predicted lower weight and head circumference at 1 year.

*Conclusion*: Children with CHD are at an elevated risk of growth faltering. HF and SGA appear to contribute to growth faltering, highlighting the importance of early recognition and targeted interventions.

**Table:** 

**What is Known** *Children with congenital heart disease (CHD) are at increased risk for growth faltering, but the independent effect of CHD is not well established.*	
**What is New** *This twin study aims to control genetic and environmental factors, showing that CHD is associated with lower growth and an increased risk of growth faltering. Among affected twins, growth outcomes also vary by disease and clinical characteristics.*

**What is Known**

*Children with congenital heart disease (CHD) are at increased risk for growth faltering, but the independent effect of CHD is not well established.*

**What is New**

*This twin study aims to control genetic and environmental factors, showing that CHD is associated with lower growth and an increased risk of growth faltering. Among affected twins, growth outcomes also vary by disease and clinical characteristics.*

## Introduction

Congenital heart disease (CHD) is the most prevalent type of congenital anomaly [1]. Hemodynamic instability from CHD affects not only nutritional status but also growth-related bodily systems, including endocrine function and musculoskeletal development [2, 3]. Even after surgical correction, residual heart failure or complications may hinder optimal somatic growth, making growth faltering common in children with CHD [4–6].

Growth faltering, defined as inadequate growth or an inability to sustain growth [7, 8], reflects a child’s current health and predict both short and long-term clinical outcomes [4, 9, 10]. However, understanding the genuine relationship between CHD and growth is difficult due to the multifactorial nature of somatic growth. Influencing factors include genetics, environment, and individual factors, such as individual health conditions [11, 12]. Genetic factors account for approximately 70–80% of population variation in height and 60–80% of weight [13], while environmental influences, such as nutrition and socioeconomic status, also play critical roles [14].

Given these influences, this study aimed to clarify the effect of CHD on growth by controlling for both genetic and environmental factors. To do this, we compared somatic growth and growth faltering in monozygotic twin pairs where one twin had CHD and the other did not. Because monozygotic twins share the same genetic makeup and are raised in similar environments, this design allows for isolation of the effect of CHD itself. Additionally, we examined whether growth varied among affected twins based on major characteristics of CHD.

## Methods

### Study design

This retrospective study used data from a referral hospital in South Korea. After receiving approval from the hospital’s Institutional Review Board (IRB), we accessed the fetal treatment center registry to identify eligible monozygotic twin pairs through chart review. Inclusion criteria were: (a) birth between 2008 and 2019, (b) confirmed monozygosity, and (c) only one twin diagnosed with CHD by a pediatric cardiologist. Exclusion criteria included: (a) both twins having chronic conditions (e.g., cardiac, renal, hepatic, endocrine disorders, or genetic/chromosomal anomalies), (b) death of one twin, or (c) CHD classified as a minor defect such as patent ductus arteriosus (PDA) or patent foramen ovale (PFO). Anthropometric measurements were collected from birth to 3 years of age to assess growth trajectories. As all data were fully de-identified, the University of Iowa’s IRB determined the study to be exempt from review.

### Data collection

Data were collected from two primary sources: (a) medical records, and (b) the Medical Examination for infants and Young Children (MEIYC) program. Through retrospective review, we obtained demographic, birth, and disease-related information, along with anthropometric data at birth from medical records. Anthropometric measurements from 1 to 3 years of age were extracted from the MEIYC program databased and prioritized to ensure consistency between twin pairs. For twins with CHD, anthropometric data from medical records were used only when MEIYC data were unavailable because of surgery, hospitalization, or other circumstances. The collected variables are described in detail below.

Anthropometric variables including weight, height, and head circumference (HC). HC was available at ages 1, 2, and 3 years only. All measurements were converted to z-scores using the World Health Organization (WHO) Anthro software. For birth and the first year of life, gestational age–adjusted growth charts were used, including corrected age for preterm infants. For ages 2 and 3 years, standard age-based growth charts were applied. Growth faltering subtypes were defined using WHO-recommended cut-off score (z < −2): underweight (weight for age z-score, WAZ), stunting (height for age z-score, HAZ), wasting (weight for height z-score, WHZ), and microcephaly (head circumference for age z-score, HCZ). Although these indicators are typically used to assess undernutrition, in this study, they were applied to evaluate growth trajectories. Stunting reflects chronic growth restriction, wasting indicates acute malnutrition, and underweight is a general marker of poor growth, possibly resulting from stunting, wasting, or both. Children who met criteria for any of the three subtypes (underweight, stunting, or wasting) were classified as having growth faltering. Additionally, to assess the intra-pair growth differences, we subtracted the z-scores of unaffected twins (non-CHD group) from those of the affected twins (CHD group). Negative values indicated growth lag in the affected twins.

Disease characteristics included cyanosis, pulmonary artery hypertension (PAH), and heart failure (HF). Cyanosis was defined as preoperative average oxygen saturation < 90% on three different occasions or documentation in the medical record. PAH was defined as mean pulmonary artery pressure (mPAP) ≥ 20 mmHg or a pulmonary vascular resistance index (PVRI) ≥ 3 WUm^2^, measured after 3 months of age. Based on the presence or absence of cyanosis and PAH, twins with CHD were grouped into four categories: CP (cyanosis with PAH), Cp (cyanosis without PAH), cP (acyanosis with PAH), and cp (acyanosis without PAH). HF was defined by at least one of the following: (a) documented diagnosis of HF in a clinician’s note, (b) a New York Heart Association (NYHA) or Ross classification of class II or higher, and (c) ejection fraction (EF) below 50% on echocardiography [15].

### Analysis

Statistical analyses were conducted utilizing SAS software version 9.4 (SAS Institute, Cary, NC, USA). Descriptive statistics (mean, standard deviation for continuous variables; frequencies and percentages for categorical variables) were calculated. Prior to analysis, the distribution of continuous variables was assessed for normality using the Shapiro–Wilk test, and equality of variances was evaluated using Levene’s test to ensure the assumptions of parametric tests were met. Paired t-test was applied to assess mean differences in anthropometric measures between CHD group and non-CHD group. To examine longitudinal differences in growth trajectories between twins, mixed-effects regression models were employed. Within the CHD group, one-way ANOVA and independent t-tests were conducted to examine whether specific disease characteristics, including cyanosis, PAH, and HF, were associated with anthropometric outcomes. For multiple subgroup comparisons, post hoc corrections were applied. Multiple linear regression analyses were performed to identify predictors of anthropometric outcomes. However, because this study relied on existing data, our ability to control data quality, including missing values, was limited. Missing anthropometric outcomes were addressed using an interpolation method to estimate values within the observation range and create a more complete dataset. A p-value of less than 0.05 was considered statistically significant for all tests.

## Results

A total of 41 pairs of twins were included in the analysis. The age of participants at the time of data collection was 89.6 ± 31.8 months (range, 40.5–161.5 months). Most were born prematurely (78.1%), and small for gestational age (SGA) was more common in the CHD group (*p* = 0.008). The mean age at corrective surgery was 13.9 ± 16.6 months, with 70.7% undergoing primary surgical correction. HF occurred in 70.7% of affected twins. Biventricular physiology was observed in 82.9%, while 17.1% had single ventricular physiology. The characteristics of the study subjects are provided in Table 1. The amount of missing data for the anthropometric outcomes at 1 year was minimal. Specifically, there were no missing data for weight, height, and head circumference data were missing for 6 participants (14.6%). These small amounts of missing data are unlikely to have influenced the results of the analyses.

**Table 1:** Demographic and Clinical Characteristics of Monozygotic Twins with One Having Congenital Heart Disease

Birth data (twin pairs)	Number of twins pairs (%)	Mean ± SD
Maternal age at delivery (years)	41 (100%)	32.4 ± 3.7
Gestational age (weeks)	41 (100%)	35.4 ± 2.1
Prematurity ^a^	32 (78.1%)	
Small for gestational age ^b^		
CHD twins	19 (43.3%)	-
Non-CHD twins	7 (17.1%)	-
Sex twin pair		
Male	15 (36.6%)	-
Female	26 (63.4%)	-
Disease characteristics of CHD twins	Number of affected twins (%)	Mean ± SD
Age at corrective surgery (months)	41 (100%)	13.9 ± 16.6
Oxygen saturation ^c^ (%)	41 (100%)	89.7 ± 10.0
Surgical correction		
Primary	29 (70.7%)	-
Staged	12 (29.3%)	-
Corrective surgery completed < 1 year of age		
Yes	21 (51.2%)	-
No	20 (48.8%)	-
Cyanotic and PAH		
Cyanosis with PAH (CP)	11 (26.8%)	-
Cyanosis without PAH (Cp)	14 (34.1%)	-
Acyanosis with PAH (cP)	9 (22.0%)	-
Acyanosis without PAH (cp)	7 (17.1%)	-
Heart failure		
Yes	29 (70.7%)	-
No	12 (29.3%)	-
Ventricular physiology		
Biventricular (BV)	34 (82.9%)	-
Single ventricular (SV)	7 (17.1%)	-
Diagnosis of CHD twins	n (%)	
Biventricular physiology (*n* = 34)		
Ventricular septal defects (VSD)	9 (22.0%)	
Tetralogy of Fallot (ToF)	6 (14.6%)	
Atrial septa defects (ASD)	4 (9.8%)	
Pulmonary atresia (PA)	4 (9.8%)	
Coarctation of the aorta (CoA)	2 (4.9%)	
Atrioventricular septal defect (AVSD)	2 (4.9%)	
Double outlet right ventricular (DORV)	2 (4.9%)	
Tricuspid valve dysplasia/atresia	2 (4.9%)	
Tetralogy of Fallot with pulmonary atresia (TOF with PA)	1 (2.4%)	
Taussig-Bing anomaly	1 (2.4%)	
Pulmonary valve stenosis	1 (2.4%)	
Single ventricular physiology (n = 7)		
Tricuspid atresia with right ventricular hypoplasia	3 (7.3%)	
Dextro-transposition of great arteries (d-TGA)	2 (4.9%)	
Corrected transposition of great arteries (ccTGA)	1 (2.4%)	
Double outlet right ventricular (DORV) with mitral atresia	1 (2.4%)	

PAH = pulmonary artery hypertension; Percentages do not sum to 100% due to rounding; p-value was calculated by Fisher’s exact test

^a^ Gestational age less than 37 weeks

^b^ If the birth weight, relative to gestational age, falls below the 10th percentile

^c^ Oxygen saturation% is a mean value of three measurements before corrective surgery

### Comparison of anthropometric measurements in twins

*Weight.* From birth, WAZ was lower in the CHD group compared to the non-CHD group (*p* = 0.046). This difference became more pronounced at 1 year of age (WAZ difference: 1.7 ± 1.6, *p* < 0.001) and persisted through 3 years, with no evidence of catch-up growth (WAZ difference: −1.3 ± 1.6, *p* < 0.001).

*Height.* HAZ was also lower in the CHD group at birth compared to the non-CHD group (−0.3 ± 1.2 vs.0.2 ± 1.1, respectively, *p* = 0.014). This disparity widened at 1 year of age (HAZ difference: −1.3 ± 1.4, *p* < 0.001), then slightly decreased at 2 years (HAZ difference: −0.9 ± 1.1, *p* < 0.001) and remained to age 3.

*Weight for Height.* At birth, both groups had WHZ below the population mean, with no significant difference between them (*p* = 0.194). By 1 year, WHZ decreased notably in the CHD group (from −0.4 ± 0.8 to −1.2 ± 1.4), while the non-CHD group showed improvement (from −0.5 ± 1.0 to −0.0 ± 1.2). The disparity remained significant at 3 years (WHZ difference: −0.9 ± 1.5, *p* < 0.001), indicating persistent growth deficits in the CHD group.

**Fig. 1:**
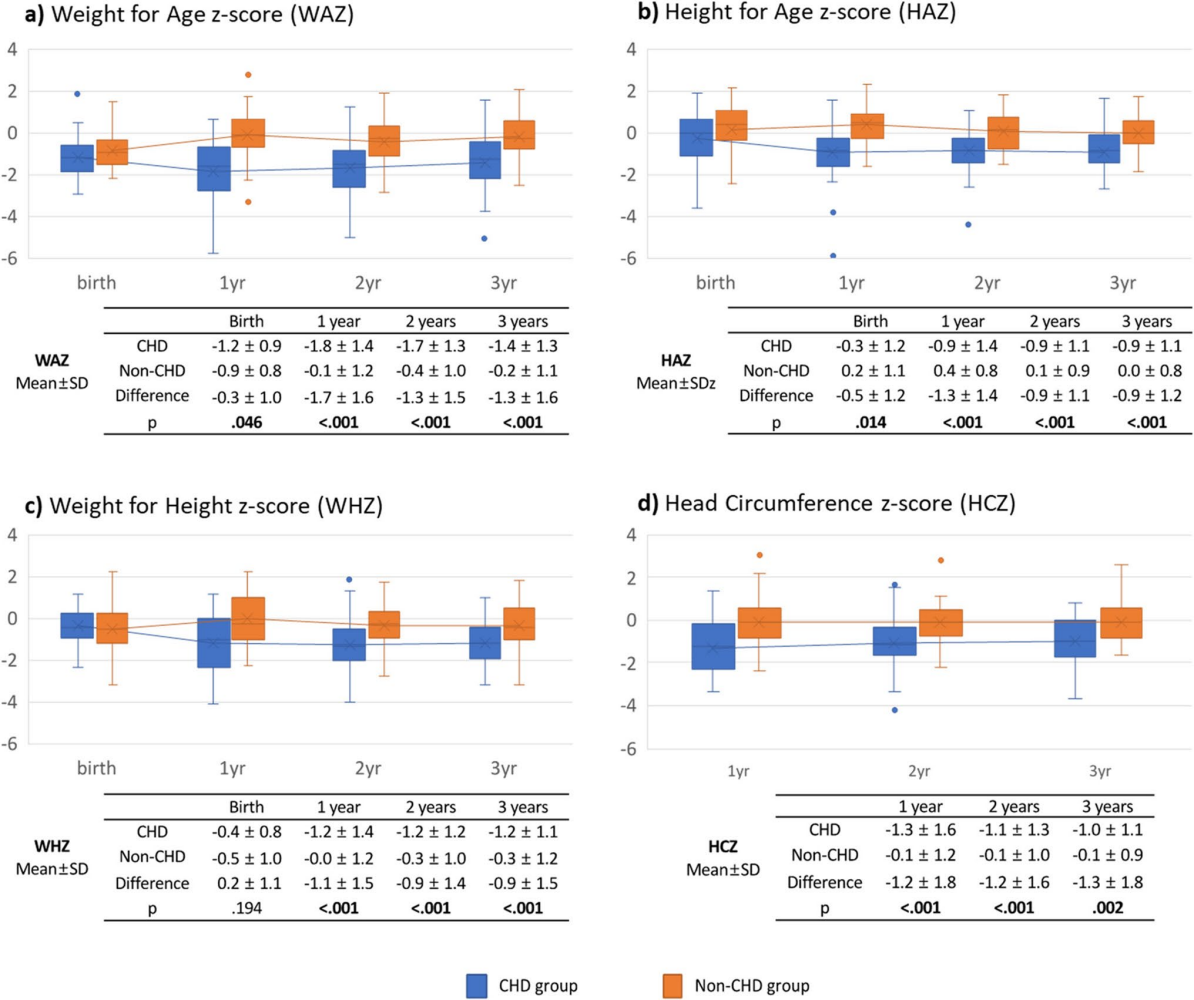
Anthropometric Measurements of Both Twin Groups (with and without CHD)

*Head Circumference*. HCZ was consistently lower in the CHD group from 1 to 3 years of age. While the non-CHD group maintained HCZ near the population mean (0.1 ± 1.2 at 1 year), the CHD group remained below −1.0 at all-time points, corresponding to approximately the 15th percentile. The HCZ difference at age 3 remained significant (HCZ difference: −1.3 ± 1.8, *p* = 0.002), with no meaningful catch-up observed (Fig. 1).

### Regression estimate of anthropometric Z-score in monozygotic twins

Mixed-effect regression analyses examining growth outcomes in monozygotic twins are presented in Table 2. For WTZ, CHD was strongly associated with lower WTZ (β = −0.832, *p* < 0.001), indicating that twins with CHD weighed less than their unaffected twin siblings. The significant negative age-CHD interaction (β = −0.017, *p* < 0.05) suggests that the weight deficit in twins with CHD increased slightly as they aged.

**Table 2:** Mixed-effect Regression Estimate of Anthropometric Z-score in Monozygotic Twins

	WTZ				HTZ				WHZ				HCZ			
			95% CI				95% CI				95% CI				95% CI	
Predictor	Beta	SE	Upper	Lower	Beta	SE	Upper	Lower	Beta	SE	Upper	Lower	Beta	SE	Upper	Lower
(Intercept)	−0.632 ***	0.163	−0.951	−0.312	0.297 *	0.149	0.005	0.589	−0.322 *	0.153	−0.620	−0.023	−0.056	0.264	−0.571	0.460
Age	0.014 *	0.006	0.003	0.025	−0.008	0.005	−0.018	0.002	0.001	0.006	−0.010	0.013	0.002	0.009	−0.021	0.015
CHD	−0.832 ***	0.176	−1.175	−0.488	−0.790 ***	0.161	−1.104	−0.476	−0.341	0.184	−0.701	0.019	−1.426 ***	0.326	−2.062	−0.790
Age:CHD	−0.017 *	0.008	−0.032	0.001	−0.006	0.007	−0.021	0.008	−0.021 *	0.008	−0.037	0.004	0.019	0.013	−0.006	0.044
R^2^/ Pseudo R^2^	Marginal: 0.20, Conditional: 0.47				Marginal: 0.17, Conditional: 0.45				Marginal: 0.11, Conditional: 0.30				Marginal: 0.16, Conditional: 0.51			

WAZ = weight for age z-score; HAZ = height for age z-score; WHZ = weight for height z-score; HCZ = head circumference z-score; CHD = presence of congenital heart disease

*** *p* < 0.001; ** *p* < 0.01; * *p* < 0.05

For HTZ, presence of CHD was similarly associated with reduced height (β = −0.790, *p* < 0.001). The age-CHD interaction was not significant, indicating that linear growth trajectories over time were parallel between affected and unaffected twins.

For WHZ, twins with CHD had lower WHZ (β = −0.341, *p* < 0.05), suggesting a tendency toward poorer weight relative to their height. The age-CHD interaction was also significant (β = −0.021, *p* < 0.05), indicating that this disparity became slightly more pronounced with age.

For HCZ, CHD had a negative effect (β = −1.426, *p* < 0.001), indicating that affected twins had smaller HC than their unaffected twin siblings. However, the age-CHD interaction term was not significant. Overall, these regressions demonstrate that CHD is independently associated with deficits in weight, height, and head circumference growth, while differences in height, weight for height, and head circumference differences remain relatively stable with age.

### Predictors of anthropometric measurements at 1 year

During the observation period, the growth z-scores of the CHD group consistently lagged behind the population average, with the most significant differences noted at 1 year of age. To investigate potential differences in growth based on disease characteristics within the CHD group, we conducted further analyses using anthropometric data at 1 year of age (Table 3). All anthropometric measurements, including WAZ, HAZ, WHZ, and HCZ, did not differ significantly based on the presence of cyanosis or PAH (p > 0.05). However, CP group tended to have lower WAZ and WHZ compared to other groups, and the cyanotic groups (CP and Cp) showed lower HAZ and HCZ than the acyanotic groups (cP and cp). Similarly, no significant differences were observed based on surgery type (primary correction vs. staged operation) or ventricular physiology (BV vs. SV) across any anthropometric indices (p > 0.05). However, children with post-operative HF had significantly lower WAZ (−2.6 ± 1.6 for the HF group vs. −1.5 ± 1.2 for the non-HF group, *p* = 0.021) and HAZ (−1.7 ± 1.7 for the HF group vs. −0.6 ± 1.1 for the non-HF group, *p* = 0.023) compared to those without HF, while WHZ and HCZ did not differ significantly by HF status.

**Table 3:** Anthropometric Measurements According to the Disease Characteristics in One Twin with Congenital Heart Disease (CHD group) at 1 Year of Age

		WAZ		HAZ		WHZ		HCZ	
Group	n (%)	Mean ± SD	p	Mean ± SD	p	Mean ± SD	p	Mean ± SD	p
Cyanosis, PAH ^1^			.433		.588		.748		.515
CP	11 (26.8%)	−2.32 ± 1.23		−1.19 ± 1.26		−1.59 ± 1.39		−1.32 ± 1.54	
Cp	14 (34.1%)	−1.73 ± 1.42		−1.11 ± 1.09		−1.14 ± 1.71		−1.67 ± 1.85	
cP	9 (22.0%)	−1.84 ± 1.78		−0.85 ± 2.07		−1.09 ± 0.97		−0.69 ± 0.95	
cp	7 (17.1%)	−1.30 ± 1.07		−0.33 ± 1.03		−0.92 ± 1.12		−1.03 ± 1.55	
Surgery type			.677		.685		.748		.341
Primary correction	29 (70.7%)	−1.87 ± 1.45		−0.88 ± 1.57		−1.17 ± 1.28		−1.10 ± 1.37	
Staged operation	12 (29.3%)	−1.77 ± 1.33		−1.08 ± 0.77		−1.32 ± 1.63		−1.61 ± 1.92	
Heart failure			**.021**		**.023**		.501		.786
Yes	12 (29.3%)	**−2.62 ± 1.64**		**−1.69 ± 1.68**		−1.44 ± 1.32		−1.44 ± 1.43	
No	29 (70.7%)	**−1.52 ± 1.18**		**−0.63 ± 1.12**		−1.12 ± 1.40		−1.28 ± 1.63	
Ventricle physiology			.264		.923		.800		.877
Biventricular (BV)	34 (82.9%)	−1.95 ± 1.47		−0.85 ± 1.48		−1.24 ± 1.31		−1.34 ± 1.68	
Single ventricular (SV)	7 (17.1%)	−1.30 ± 0.92		−0.89 ± 0.70		−1.09 ± 1.72		−1.23 ± 0.88	

PAH = pulmonary artery hypertension; CP = Cyanosis with PAH; CP = Cyanosis without PAH; cP = Acyanosis with PAH; cp = Acyanosis without PAH; WAZ = weight for age z-score; HAZ = height for age z-score; WHZ = weight for height z-score; HCZ = head circumference z-score

P-values are based on independent t-tests

^1^ One-way ANOVA used

Values significant at *p* <.05 are in bold

Multiple linear regression analyses were performed to identify predictors of anthropometric z-scores at 1 year. In the full models, none of the predictors were statistically significant, although SGA showed a negative association with WAZ, HAZ, and HCA. In the best AIC models, SGA remained a significant predictor of lower WAZ (β = –0.87, 95% CI –1.72 to –0.02, *p* < 0.05) and HCZ (β = –0.87, 95% CI –1.72 to –0.02, *p* < 0.05), while staged surgery had modest, non-significant associations with HCZ. Other clinical characteristics, including PAH, ventricular physiology, cyanosis, HF, were not significantly associated with anthropometric z-score. Overall, SGA was the most consistent determinant of impaired growth at 1 year among twins with CHD (Table 4).

**Table 4:** Multiple Linear Regression Estimate of Anthropometric Z-score at 1 year in Twins with CHD

Variable	WTZ				HTZ				WHZ				HCZ			
			95% CI				95% CI				95% CI				95% CI	
	Beta	SE	Lower	Upper	Beta	SE	Lower	Upper	Beta	SE	Lower	Upper	Beta	SE	Lower	Upper
**Full model**																
(Intercept)	−0.508	1.049	−2.642	1.627	−0.646	1.009	−2.698	1.406	−0.277	1.082	−2.478	1.923	0.813	1.227	−1.704	3.330
SGA	−0.669	0.490	−1.665	0.326	−0.834	0.471	−1.791	0.123	0.183	0.505	−0.843	1.209	−1.013	0.588	−2.220	0.193
PAH	−0.402	0.445	−1.308	0.504	−0.111	0.428	−0.982	0.760	−0.332	0.459	−1.266	0.602	0.449	0.535	−0.649	1.546
Biventricular	−0.726	0.935	−2.627	1.175	0.175	0.898	−1.653	2.003	−1.020	0.963	−2.980	0.940	−1.426	1.104	−3.691	0.839
Cyanosis	−0.334	0.545	−1.442	0.774	−0.099	0.523	−1.164	0.966	−0.702	0.561	−1.844	0.439	−0.421	0.661	−1.776	0.934
HF	−0.246	0.550	−1.365	0.874	−0.530	0.529	−1.607	0.546	0.758	0.567	−0.396	1.912	−0.208	0.648	−1.537	1.120
Female	0.418	0.458	−0.513	1.349	0.794	0.440	−0.101	1.688	0.078	0.472	−0.881	1.038	0.248	0.591	−0.858	1.354
Staged surgery	−0.368	0.695	−1.782	1.046	−0.187	0.668	−1.546	1.172	−0.639	0.716	−2.096	0.819	−1.599	0.838	−3.317	0.119
Num. Obs	41				41				41				35			
R^2^	0.189				0.222				0.095				0.292			
Adj. R^2^	0.016				0.057				‑0.097				0.109			
AIC	152.5				149.2				155.0				135.3			
**Best AIC**																
(Intercept)	−1.439 ***	0.288	−2.021	−0.858	−1.108 **	0.396	−1.909	−0.306	−1.211 ***	0.214	−1.643	−0.780	−0.526	0.374	−1.288	0.236
SGA	−0.869 *	0.422	−1.723	−0.015	−0.740	0.405	−1.560	0.079	-	-	-	-	−1.226 *	0.495	−2.241	−0.211
Female	-	-	-	-	0.804	0.419	−0.044	1.652	-	-	-	-	-	-	-	-
Staged surgery	-	-	-	-	-	-	-	-	-	-	-	-	−0.960	0.546	−2.071	0.152
Num. Obs	41				41				41				35			
R^2^	0.098				0.171				0.000				0.199			
Adj. R^2^	0.075				0.128				0.000				0.149			
AIC	144.8				141.8				145.0				129.7			

WTZ = weight-for-age z-score; HTZ = height-for-age z-score; WHZ = weight-for-height z-score; HCZ = head circumference-for-age z-score; SGA = small for gestational age; PAH = pulmonary artery hypertension; HF = heart failure

*** *p* < 0.001; ** *p* < 0.01; * *p* < 0.05

## Discussion

The primary aim of this study was to evaluate the relationship between CHD and growth faltering while controlling the influence of genetic and environmental factors. To achieve this, we conducted a longitudinal comparison of the growth of monozygotic twins, where one twin had CHD, and the other did not.

At birth, more children in the CHD group were SGA than in the non-CHD group (46.3% vs. 17.1%, *p* = 0.008). The placenta plays a crucial role in fetal growth, and both the fetal heart and placenta develop interdependently. Placental dysfunction can hinder cardiac cell proliferation and angiogenesis, contributing to the development of CHD [16]. Consequently, newborns with CHD are at a higher risk of intrauterine growth restriction, including low birth weight and SGA. Prior studies have similarly demonstrated that fetuses with CHD are more than twice as likely to be SGA or low birth weight [17–19]. The high prevalence of SGA in the CHD group suggests that CHD may have impacted prenatal growth.

From one year of age onward, all anthropometric measurements in the CHD group were significantly lower than those in the non-CHD group. While the non-CHD group demonstrated steady improvement in growth z-scores over time, the CHD group experienced a relative decline in all anthropometric z-score compared to birth. Mixed effect regression analyses demonstrated significant associations between CHD and growth faltering across WTZ, HTZ, WHZ, and HCZ, with age and CHD interactions significant for WTZ and WHZ, indicating that growth disparities between twins became more pronounced with increasing age. Previous studies in healthy twins raised in the same environment have shown minimal differences in body size [20, 21]. These findings suggest that CHD is associated with poorer growth trajectories, although the specific biological mechanisms could not be directly examined.

Notably, the most pronounced growth disparities and the highest prevalence of growth faltering in the CHD group observed during the first year of life. This is consistent with prior research indicating that infants with CHD, especially in the preoperative period, experience significant growth restrictions. For instance, a longitudinal study showed that growth declined prior to cardiac surgery but improved postoperatively [22]. Other studies have similarly reported higher prevalence of underweight, stunting, and wasting in children under 12 months compared to those aged 1–5 years [23]. Surgical correction may improve growth by stabilizing hemodynamics, decreasing energy expenditure, and enhancing nutrient intake and absorption [24–26]. Given that most cardiac surgeries occur between 6 months and 2 years, improvements observed during this period may be due in part to symptom resolution and improved cardiovascular function following surgery. In our study, although anthropometric measures in the CHD group showed slight improvement with age, they remained consistently lower than those of their twin siblings, with no evidence of full catch-up growth. These persistent deficits highlight the enduring impact of CHD on long-term growth and emphasize the need for ongoing monitoring and individualized intervention to support optimal growth.

Weight growth appeared to be more adversely affected than height growth in the CHD group. WHZ declined substantially over time, indicating that the CHD group did not gain adequate weight relative to their height. This may be related to evidence that weight is more sensitive to acute health and environmental factors, whereas height is primarily influenced by chronic and genetic conditions [27]. Research suggests that 70–80% of height and 60–80% of weight or body mass index (BMI) variability is genetically determined [13]. As such, weight growth may be more vulnerable to non-genetic factors, such as CHD. HC was also consistently lower in the CHD group, with the most pronounced deficit at 1 year and only modest improvement by age 3. Prior literature links factors, such as prolonged intensive care unit stays, sedative exposure, and frequent hospitalizations to impaired brain growth [28, 29]. Impaired brain development can lead to delays in motor coordination, physical function, and language skills [4, 30, 31]. In a study of infants with developmental deficit, decreased HC growth velocity has been associated with developmental deficit and motor delay [32]. Given that early childhood is a critical window for brain development, and that HC is a widely used clinical proxy for brain growth, continuous monitoring of HC in children with CHD is essential, even if changes appear more subtle than those seen in weight or height.

Within the CHD group, growth outcomes varied according to disease and clinical characteristics. Cyanosis has been widely implicated in growth faltering due to increased resting energy expenditure, intestinal mucosal injury, and impaired nutrient absorption [33–35]. In addition, PAH are associated with breathlessness, fatigue, and exercise limitation, which can lead to an imbalance in energy expenditure and consequently can exacerbate the risk of growth faltering [9]. In our findings, children with both cyanosis and PAH (CP group) exhibited poorer growth in weight, height, and head circumference compared to those without these conditions (cp group). Although the differences did not reach statistical significance, this growth difference relatively better growth outcomes in the absence of significant hemodynamic complications. Importantly, HF was significantly associated with poorer growth. Children with HF had lower WAZ and HAZ compared to those without HF. HF is known to affect nutritional status through multiple mechanisms such as reduced appetite, feeding intolerance, increased metabolic demand due to elevated cardiac workload, and impaired nutrient absorption and utilization [36–38]. These findings underscore the need for early identification and proactive nutritional interventions in children with CHD and HF. Regarding ventricular physiology, children with BV physiology had lower weights than those with SV physiology, but this was not statistically significant. Prior studies have reported mixed findings. Although TengShu [39] reported significantly impaired growth in children with complex biventricular physiology, other studies did not find significant differences between SV and BV groups [9, 40, 41]. Conversely, DaymontNeal [42] found that children with SV defects had lower overall anthropometric measurements than those with BV defects. In our study, children with minor defects were excluded, and BV diagnoses included moderate to severe types of CHD, such as tetralogy of Fallot (with/without pulmonary atresia), double outlet right ventricle, and tricuspid atresia. This broader inclusion of complex CHD within the BV group may explain the lack of significant growth differences between BV and SV groups. Similarly, surgical type (primary correction vs. staged repair) was not significantly associated with growth outcomes. In the regression analyses, other clinical factors, including PAH, ventricular physiology, and cyanosis, were also not independently significant predictors. Only SGA was a significant predictor of WAZ and HCZ, while staged surgery and female sex showed modest, non-significant association with growth outcomes.

Overall, our findings indicate that CHD is associated with growth faltering trajectories, with SGA status and HF showing the association in this cohort. Recent meta-analysis shows that growth-restricted infants with CHD are at heightened risk for adverse neurodevelopmental outcomes, suggesting that growth faltering may reflect broader developmental vulnerability rather than a purely nutritional challenge [43]. Accordingly, growth faltering may serve not only as markers of nutritional status but also as early indicators of neurodevelopmental risk. These findings underscore the importance of proactive monitoring and targeted strategies to optimize growth in children with CHD, with the goal of supporting both somatic and developmental outcomes. Although our result provides evidence of an association between CHD and growth faltering, it does not identify the specific biological mechanisms underlying these associations. Further study is needed to explore the physiological pathway linking CHD, altered energy balance, and growth, as well as the potential impact of surgical timing, postoperative recovery, and nutritional interventions on growth trajectories in children with CHD.

### Limitation

Several limitations of this study should be considered when interpreting the findings. First, the retrospective nature of the study limits the ability to establish causal relationships and may introduce unmeasured confounding factors. Data collection was restricted to information available in existing data, which may not fully capture all relevant variables influencing growth outcomes. Second, this study relied primarily on anthropometric measures to evaluate the influencing of CHD on somatic growth. While clinically meaningful, these measures do not provide insight into the underlying biological mechanisms. Further studies using cardiac biomarkers, energy expenditure, and hormonal profiles could offer a more comprehensive understanding of how disruptions in cardiovascular physiology and endocrine regulation interact to influence growth faltering in children with CHD. Third, the decision to exclude twin pairs in which both had chronic conditions may have introduced selection bias. This exclusion could have eliminated cases in which undiagnosed or shared factors influenced both the development of CHD and somatic growth, potentially underestimating the true complexity of relationship between these factors. Additionally, although the twin design controls for shared environment and genetics, it does not isolate the effects of CHD from the necessary treatments and consequences associated with the condition. Lastly, the study is limited by its single-center, single-country design and follow-up restricted to 3 years, highlighting the need for larger, multicenter, and longer-term evaluations.

## Conclusions

Despite these limitations, our study highlights the potential impact of CHD on somatic growth by comparing growth between monozygotic twins—one with CHD and one without. From 1 year of age onward, children with CHD exhibited reduced growth in height, weight, and head circumference compared to their unaffected twin siblings, with no evidence of complete catch-up growth during the observation period. Growth faltering was particularly pronounced in children with HF, underscoring the importance of targeted intervention. PAH and cyanosis may further exacerbate growth challenges by increasing metabolic demand and hindering energy absorption, although its independent effect was not significant in our cohort. Overall, these findings emphasize the vulnerability of children with CHD to growth faltering and the need for proactive monitoring, nutritional support, and individualized management strategies to optimal growth and long-term health outcomes. Future studies with larger sample size and more specified physiological assessments are warranted to clarify the mechanisms linking specific CHD disease characteristics, including HF and PAH to growth faltering.

## Data Availability

No datasets were generated or analysed during the current study.

## Abbreviations

CHD: Congenital Heart disease
WAZ: Weight for age z-score
HAZ: Height for age z-score
WHZ: Weight for height z-score
HCZ: Head circumference z-score
PAH: Pulmonary artery hypertension
HF: Heart failure
BV: Biventricular
SV: Single ventricular

## Declarations

The authors declare that no funds, grants, or other support were received during the preparation of this manuscript.
